# Rethinking Social Interaction: Empirical Model Development

**DOI:** 10.2196/18558

**Published:** 2020-04-23

**Authors:** Jone Bjornestad, Christian Moltu, Marius Veseth, Tore Tjora

**Affiliations:** 1 Department of Social Studies Faculty of Social Sciences University of Stavanger Stavanger Norway; 2 Department of Psychiatry District General Hospital of Førde Førde Norway; 3 Department of Clinical Psychology University of Bergen Bergen Norway

**Keywords:** social interaction, social functioning, social media, model, empirical, adolescence, health science

## Abstract

**Background:**

Social media is an integral part of human social life. More than 90% of young people use social media daily. Current theories, models, and measures are primarily based on face-to-face conceptions, leaving research out of sync with current social trends. This may lead to imprecise diagnoses and predictions.

**Objective:**

To develop a theoretically based empirical model of current social interfaces to inform relevant measures.

**Methods:**

A three-stage, qualitative, data-collection approach included anonymous individual Post-it notes, three full-class discussions, and 10 focus groups to explore 82 adolescents’ relational practices. Data analysis followed a meaning-condensation procedure and a field-correspondence technique.

**Results:**

We developed an empirical model that categorizes adolescents’ social interactions into five experiential positions. Four positions result from trajectories relating to social media and face-to-face social interaction. Positions are described by match or mismatch dynamics between preferred and actual social platforms used. In matched positions, individuals prefer and use both face-to-face and social media platforms (position 1), prefer and use face-to-face platforms (position 2), or prefer and use social media platforms (position 3). In mismatched positions, individuals prefer face-to-face interactions but use social media platforms (position 4) or prefer social media but use face-to-face platforms (position 5). We propose that matched positions indicate good social functioning while mismatched positions indicate serious social challenges.

**Conclusions:**

We propose a model that will expand previous unidimensional social interaction constructs, and we hypothesize that the described match and mismatch analyses provide conceptual clarity for research and practical application. We discuss prediction value, implications, and model validation procedures.

## Introduction

Social media platforms are technology-mediated tools that enable individuals to create, share, and exchange ideas, images, and information through online communities and networks [1,2]. Social media has become an integral part of current social life and provides new opportunities for accommodating the core human need of being emotionally affiliated with a community [3]. Globally, there are approximately three billion registered social media profiles, and the number of social media platforms is growing exponentially [4]. Young persons are the heaviest consumers, with more than 90% using social media on a daily basis [5]. Despite a minimum access age of 13 years for several platforms, reports suggest that 75% of 10-12-year-olds have a social media account [6,7].

Despite burgeoning social media use, current social interaction theories [8,9], models, and measures are largely based on face-to-face conceptions, resulting in an outdated understanding of how social media phenomena materialize in social life [10-14]. This may lead to lack of analytic precision across a range of settings. For example, within the field of mental health, individuals with a rich online but limited face-to-face social life could currently be assessed as having poor social functioning. This may have monumental consequences, as a false low social-functioning score could lead to a false positive psychiatric diagnosis with subsequent incorrect or excessive treatment. Furthermore, imprecise understanding of social media functioning could deprive the helper of the opportunity to map and facilitate central online coping areas [14].

Core social capacities, such as emotion regulation, attachment, language, mentalization, and agency, develop from a starting point of physical, time-synchronized, face-to-face interactions with caregivers [8,15,16]. Mature relationships gradually manifest throughout adolescence [17,18]. Use of social media as part of human social life accelerates during the same time period. This seems significant, as adolescence, due to rapid bodily, cognitive, and emotional changes, is considered a period of both great vulnerability and great potential [19]. Youths display general limitations in reflexivity, emotion regulation, and ability to consider consequences before acting, making them more easily affected by influences, both through social media and in face-to-face situations. Negative social comparisons, social exclusion, social media addictions [18], bullying, cyberbullying [20,21], and cybervictimization [22] increase chances for poor development and psychopathology. Moreover, adolescents are formed prosaically by positive grown-up role models or peers validating and teaching flexible strategies. These factors may act protectively when youths fluctuate between the group norms and identities of their peers, both on social media and in face-to-face settings [12,17,23]. Consequently, social media may be understood as adding a further layer of complexity that adolescents must master during an already complex life period.

Technological innovation affects society and human behavior in fundamental ways and changes the interfaces between individuals [24]. Specific technologies, such as social media, do not merely add to the possibilities for communication, but also change the nature of communication. At face value, when compared with face-to-face social interaction, social media platforms represent radical changes, creating possibilities for asynchronistic and multicast communication and an unlimited number of possible contacts [2,25]. Further, by removing boundaries of time, space, and language, and by adding artificial intelligence, social media makes human relationships digital.

Online social technology may raise challenges [2,12]; for example, does social media require extra social flexibility or is it adaptable to facilitate communication for persons who may experience social limitations face-to-face, such as persons with social anxiety or severe mental illness [14,26]? Among other things, distance in both time and space means that fewer bodily senses are used and gives one the ability to pause information flow [27]. Compared to face-to-face communication, these features may reduce social withdrawal but, at the same time, may also be obstacles to precise communication by decreasing information accuracy. Social media also makes misinformation and use of several or false identities easier and more common [28].

Valid research into human social behavior can only be achieved with clear operationalizations integrating contemporary social processes. To achieve this, incorporating and investigating the added complexity that social media brings to sociality is imperative. Theories, models, and measures have largely ignored this integration [14], and the need for modification is precarious.

In this study, we implemented a large-scale, qualitative, in-depth investigation of 82 young individuals’ experiences with current social life, aiming to develop an empirically informed theoretical model of face-to-face and social media interaction.

Our research question is as follows: How do adolescents experience and practice social interaction after the added complexity brought by social media?

## Methods

### Overview

A three-stage, qualitative, data-collection approach involved anonymous individual Post-it notes, three full-class discussions, and 10 focus groups to explore 82 adolescents’ relational practices on social media. We used a reflexive thematic approach [29,30] for implementation and analyses. This study was approved by the Regional Ethical Committee (2018/2273/REK nord). Participants gave their informed written consent.

### Sample and Recruitment

Most people participate in the public school system in Norway, including people with, for example, psychological problems and disabilities [31]. Special care is free in Norway, and the result is an overall representative student population and a high number of graduating students [31]. The study sample (N=82) was recruited between February and April 2019 and was based on strategic sampling from three different high schools in Rogaland county, Norway. To approximate population representativeness, we invited schools with different socioeconomic profiles, admission requirements, and geographical localizations. The sample consisted of six school classes: three classes of students in a general higher education preparation program, two classes of students in a health and social work training program, and one class of students in an electrical craft training program.

Sample size was reviewed after four and eight focus group interviews. We stopped recruiting after 10 focus groups because we considered the last two not to have contributed additional information [32]. Participants were 71% (58/82) female and participant ages ranged from 17 to 19 years. They were admitted in either programs for university admissions or vocational training. Several students from three classes had psychology as an elective subject.

### Procedure and Data Collection

First, we used a design-thinking approach aiming to reveal individual perspectives [33-35]. We applied a “silent” Post-it note technique with the following instructions: (1) “Please make as many statements as possible, positive and negative, about what social interaction is for you, including interaction on social media” and (2) “What questions are relevant to ask about current social interaction, including social media interaction?” Then, together with the participants, we categorized Post-it notes thematically, in vivo, based on similarity in content. Using this approach had two purposes: (1) to form a starting point for full-class and focus group discussions and (2) to ensure field correspondence, give voice to outliers, and compensate for limitations in the focus group approach. These full-class introduction sessions lasted approximately 45 minutes.

Second, we organized full-class discussions. We encouraged participants to elaborate on their Post-it notes and comment on their claims on social interaction. The full-class discussions lasted approximately 60 minutes each.

Third, we divided classes into focus groups for in-depth interviews: total number of participants was 82, total number of focus groups was 10, and number of participants in each focus group ranged from 5 to 12. The participants’ primary teachers created the focus groups, as we considered them to be best suited for the task given their familiarity with the individual students and relationships among them. Focus groups were used to further elaborate on the themes raised in the Post-it notes and the full-class sessions. The researchers used this opportunity to raise in-depth questions about themes emerging from steps 1 and 2. As expected, the focus group format was more manageable for several of the study participants, resulting in increased engagement and new topics.

At the end of each focus group, participants were invited to provide any relevant information that had not yet been thematized. All steps of the investigation were implemented in classrooms at the participants’ respective high schools. Post-it notes were photographed, and full-class discussions and focus group interviews were audio recorded and transcribed verbatim for the purposes of analysis.

### Analysis

For inductive analysis, we employed a six-step reflexive thematic approach [29,30] concretized in Textbox 1. To strengthen the credibility of the study, the four researchers conducted the analytic procedure independently. During collaborative meetings, the researchers compared their interpretations, agreed on themes with accompanying quotes and model content, and validated the findings by consensus decision [36], dedicating special attention to steps 4 to 6 presented in Textbox 1. To overcome possible disagreement in the collaborative analytic meetings, we agreed on the following decision rules in the preparatory phases of the study: (1) to resolve minor disagreements by the principle of parsimoniousness and (2) to resolve major disagreements by (a) an inductive principle using the raw data as a compass, aiming to select the descriptions most closely reflecting the experience of the phenomena at issue, and (b) further applying the principle of best argument as described above.

**Steps of thematic analysis.**

Step 1. Becoming familiar with the data through thorough reading of the Post-it notes, transcribed full-class discussions, and focus group interviews, thereby forming a main impression of the experiences of the participants and identifying potentially important themes. A *theme* was defined as a verbalization capturing an important element of the data in relation to the research question, representing a patterned response in the dataset.

Step 2. Generating initial codes, which were defined as the most basic segments of the raw data that could be assessed in a meaningful way regarding the specific phenomenon. For example, the participants’ descriptions of flexibly using both face-to-face and social media platforms were given the tentative code *flexible use*.

Step 3. Searching for and developing candidate themes and subthemes. Remaining codes were set aside at this phase in a separate category for the purpose of being further analyzed and incorporated when appropriate. For example, the theme *mentalization* was initially referred to as *empathy*. However, during analysis we perceived this theme to also include the participants’ reflections of their personal contribution to the social interactions they engaged in. Hence, we altered the theme name to *mentalization*.

Step 4. Reviewing themes to develop a coherent thematic map and considering the validity of individual themes in relation to the dataset.

Step 5. Defining and naming themes: further refining and defining themes, identifying the essence of themes, identifying subthemes, and summarizing the contents of the main themes into what each researcher considered to best represent participants’ experiences. When refinements no longer added substantially to the themes, the analytic process was closed.

Step 6. Determining the relevance of a particular theme by counting the frequency of the relevant meaning units and combining this with our interpretation of how central the theme was perceived to be to the social life of the participants. It was at this point that we agreed that themes could be transferred to model positions.

## Results

**Figure 1:**
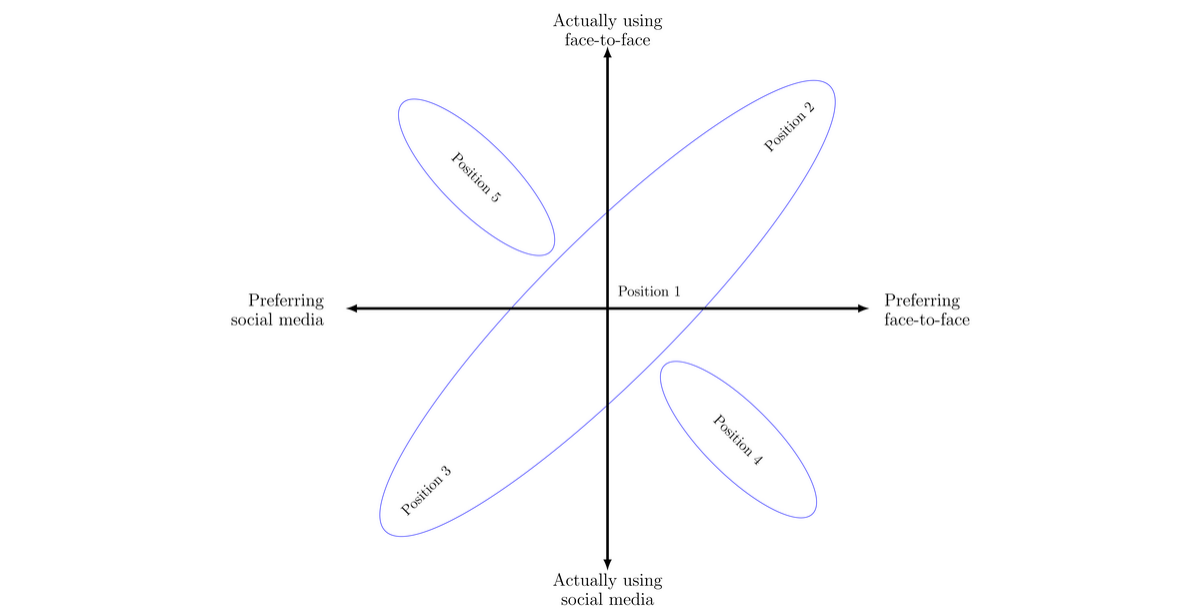
Youth social interaction positions.

**Figure 2:**
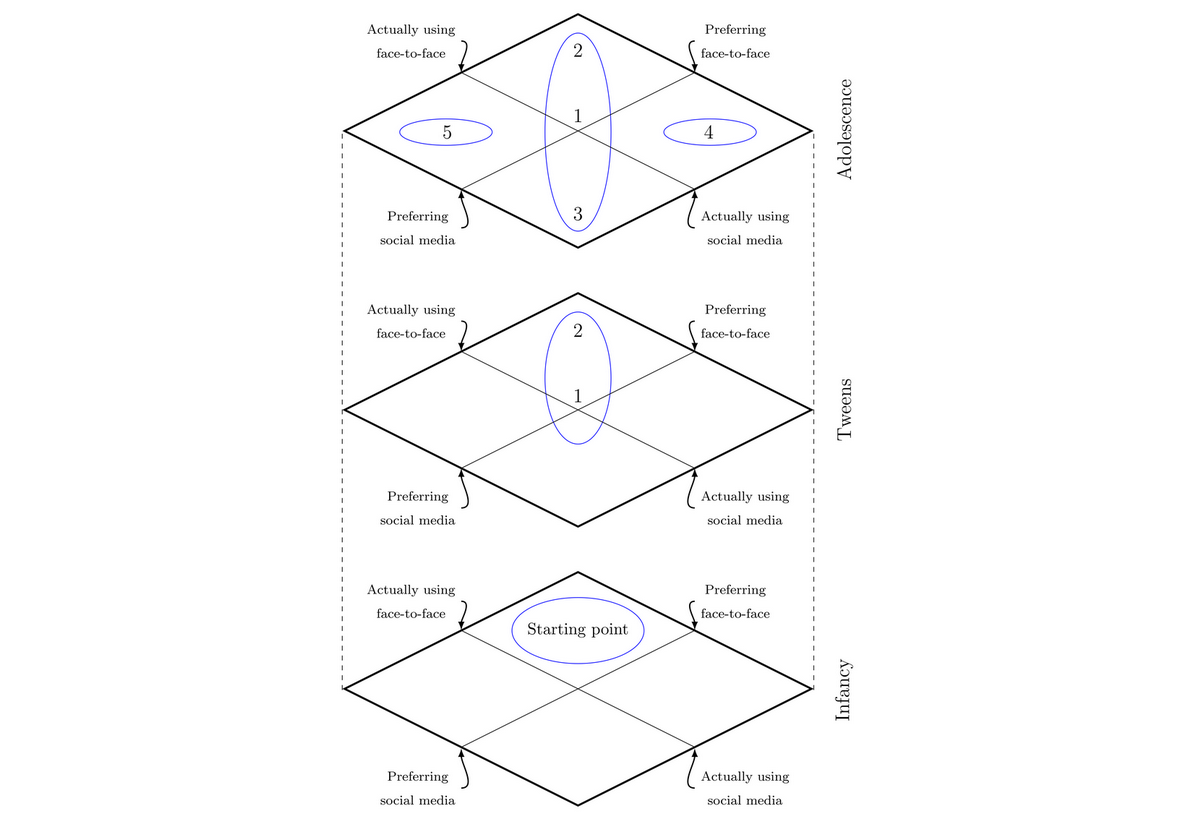
Social interaction positions: complete model.

Our analysis resulted in a theoretically informed empirical model for the integration of social media and face-to-face interaction (see Figures 1 and 2).

### Model: Suggested Social Positions for Adolescent Social Interaction

*Infancy* denotes the model’s starting point in childhood’s physical, time-synchronized, face-to-face experiences with caregivers. The second layer, *Tweens*, concerns the introduction of social media experiences, reflecting the approximate period when social media becomes a significant part of human social life [6]. The third layer, *Adolescence*, reflects the empirical analyses in this study, which suggest that adolescents’ social interactions can be understood through five social positions resulting from multiple developmental trajectories. The x-axis presents the preferred type of social interaction, while the y-axis presents the actual type of social interaction. In the participants’ experiences, face-to-face interaction and social media interaction are overlapping social formats. Hence, both x- and y-axes reflect a continuum of social interactions, not binary oppositions. For transparency, illustrations of the empirical basis for the model are presented in Table 1 and Table 2. The five social positions (see Figure 2) are detailed in the following sections.

### The Concept of Matching

In the model presentation, *match* implies correspondence in supply and demand between personal resources—social skills, social behaviors, personal values, interests, etc—and social context demands underlying social networks and relationship maintenance. All individuals in matched positions reported that they have communities, digital or analog, in which they have a sense of emotional affiliation and where they can act as independent agents. Hence, *match* refers to the individual’s ability to gain access to available social benefits; master the current social norms, game rules, and contexts; and display social functioning fulfilling their social needs. Our empirical data suggest that some individuals satisfy their social needs, and hence achieve match, solely in face-to-face settings, whereas some achieve match solely in social media settings, and some move flexibly and interchangeably between face-to-face and social media settings. These three matched positions constitute the *cigar shape* in the model (see Figure 2).

Conversely, *mismatch* between preferred and actual social platform implies lack of correspondence between personal social resources and contextual demands. Individuals experiencing mismatch experience challenges in accessing available social benefits and accommodating to social norms and game rules, and display a social functioning profile not satisfying their social needs. Our empirical data and the current literature indicate two mismatching positions: individuals who prefer face-to-face interactions but are unwillingly using social media, and individuals who prefer social media interaction but are unwillingly using face-to-face interactions.

### Position 1: Flexible Match—Preferred and Actual Interactions Are Both Face-to-Face and Via Social Media

Individuals speaking from position 1 were characterized as flexible and well-functioning individuals who are using social media and face-to-face formats in continuous adaption to contextual changes. Participants typically describe these individuals as living rich face-to-face social lives but, at the same time, actively enriching their social life with social media, for example, by initiating new relationships, seeking information and entertainment, and preserving already-established relationships. They were also perceived as good at critically assessing information quality and using their established face-to-face and online social networks to protect themselves against hazards, for example, individuals trying to exploit them or fake profiles (ie, catfishing). Position 1 appears to be the most flexible of the model positions.

### Position 2: Match—Preferred and Actual Interaction Is Face-to-Face

Individuals with a current affiliation with position 2 consider face-to-face relationships to be the only authentic relationship format and therefore seek this form of contact. Participants described individuals remaining within this position as largely respecting and upholding social norms regulating face-to-face interactions, such as honesty and respecting individual differences and personal boundaries. Norm violations result in sanctioning and ultimately social exclusion. Data suggested a value-laden conflict between positions 2 and 3 on what constitutes authenticity. This conflict was mostly addressed by individuals in position 2, who suggest that purely online social life is inferior, as expressed in statements such as “a pure online life is not a full social life” or “the goal of online contact is always physical meetings.”

### Position 3: Match—Preferred and Actual Interaction Is Via Social Media

Individuals with a sense of belonging in position 3 reported preferring social media relationships, including gaming, over face-to-face relationships. Although they used face-to-face interactions early in life, the significance of face-to-face relationships gradually decreased with age and primarily serves to meet practical and societal demands, such as attending school and family meetings; when choosing based on their own preferences, they live their lives mainly on social media. Within their social media communities, they described it as less important whether they use a nickname or real name. Nicknames were, for some, described as just as important and real as their given names. Although real names and personal information are often gradually revealed, the goal of relationships is not necessarily to evolve into physical meetings, as is the case for individuals in position 2. Position 3 social norms seem similar to norms associated with position 2, but are typically sanctioned through platform moderators.

Answering the authenticity criticism promoted by individuals in position 2, individuals speaking from position 3 were consistent in valuing social media interactions as authentic and as, for them, more appreciated than face-to-face relationships, and they described long-lasting social media relationships. These types of relationships were mainly described in the context of flexible social media platforms that allow for complex online interactions.

### Position 4: Mismatch—Preferred Interaction Is Face-to-Face; Actual Interaction Is Via Social Media

Data suggested that position 4 was taken by individuals preferring face-to-face social interaction but using social media social interaction. No participants confirmed affiliation with this position themselves. Rather, findings are based on participants’ descriptions of other individuals and their first-hand experiences with them through both face-to-face and social media interactions. These third-person descriptions involved increased distance from the phenomenon compared to the first-person descriptions of position 1, 2, and 3; that is, this position is described from an outside perspective rather than as lived experiences. Consequently, the descriptions may be affected by distance in perspective, and the risk of fundamental attribution errors increases accordingly. Nevertheless, individuals in position 4 are still judged based on their behaviors in the data material, and the consequences are negative characterizations and high risk for social exclusion.

Participants described individuals in position 4 as having limited social networks, poor social skills, and poor compliance with face-to-face and social media norms and social game play. Examples describe odd behaviors, such as contacting strangers face-to-face or online in ways more suitable for close friends, and cross-border behaviors, like communicating through fake social media profiles (ie, catfishing). Poor social skills were also described as making these individuals vulnerable for exploitation, including exploitation by individuals seeking economic, sexual, or other personal gains.

Participants described a collective effort, using each other’s face-to-face and online social networks, to identify and protect against hazards from individuals in position 4. Their main strategy was social exclusion, for example, through profile blocking. Despite describing these individuals as displaying similar behaviors in face-to-face and social media settings, face-to-face exclusion was described as more brute and absolute than social media exclusion. The nearly unlimited opportunities for new encounters were described as the main reason why position 4 individuals remained social media users, despite preferring face-to-face interaction. Nonetheless, the descriptions suggest that widespread social exclusion is a problem for this group and that they have few opportunities for realizing protracted social relationships.

### Position 5: Mismatch—Preferred Interaction Is Via Social Media; Actual Interaction Is Face-to-Face

Position 5 is descriptive of individuals who possibly prefer social media but use or are forced to engage in face-to-face social interaction. Participants, only to a limited degree, gave third-person descriptions with position 5 characteristics, suggesting that this is an outlier position. Hence, the attribution error risk is also relevant for this position. Nevertheless, based on empirical evidence for this position from other studies [37-39], we include it in the proposed model. We suggest that this position might apply to two groups of individuals: (1) those who lack social media awareness and possibilities due to, for example, limited internet access, and (2) those who, regardless of awareness, otherwise lack social media interest, have negative attitudes toward social media, or simply resist social media participation [37,38]. Individuals with limited social media skills or in disadvantaged social economic situations also plausibly belong to this group [39]. These individuals would perhaps have a more satisfactory social life if social media were integrated in their daily routines. This may be the case for several marginalized groups, such as prison inmates or individuals in other facilities where people are compulsively placed together.

**Table 1:** Experiences of social interaction across current social interfaces, by theme and position.

Theme and positions		Exemplary quotes
**Establishing and maintaining social relationships**		
	3	I use Discord a lot [complex social media platform] and for me it’s not just something that can replace Skype, but also a place they can find new communities or support groups where you can meet new friends. And it’s a place where you can, for example, if you meet a friend in a game and you want to play with them more, you can contact them on Discord and then just talk to them there or join like a server or like communities with them. And in that way keep in touch and when you use Discord as much as I do and many others do, you meet a lot of new people and you can have a lot of good friendships, even if they are just on Discord. For example, I have people I know better than my brothers who I only talk to on Discord.
	1	Because I can’t think of many friends I’ve met on social media. It’s more to keep in touch. People I don’t see that often live in other places and stuff.
	1 and 3	Yeah, for me at least, if I meet new people, then first it’s Facebook, then become friends on Facebook, and then maybe follow up on Instagram, and then when you’ve either met again or chatted on Facebook, or something like that, then you can like... You go onto SnapChat. But it’s just like... Well, that’s how I do it, in that order, kind of. And SnapChat is more, for me, when I know someone. While Facebook can, is kind of more, like general: “Ok, we’re friends on Facebook, at least, so then we can contact each other on Facebook,” if we... From there, and then develop it further to other social media, sort of.
	4	All these friend requests from people in different countries and you have no idea who they are. And they have closed information. Then you don’t accept them.
	4	When it says you have no friends in common you don’t often think “oh, I think maybe I know who they are!”
	1	So the more friends you have in common, the better the chances are.
	2	Yeah, cause the whole social network has just been moved over to social media. It’s reality moved over onto the internet. The same things that happen in reality happen there, just a bit differently.
	2	Because now I kind of have the base I need to meet people in reality, face-to-face. I don’t, like, need to go online to meet people online and then meet them in reality, kind of.
	1	Back to the thing about getting new friends. I feel like say you’re working out at the gym and you see someone and you know who they are, but you’re not brave enough to talk to them. Then social media is kind of a really good, like, ice breaker, because you can start by following them, or adding them somewhere and then that kind of opens up for a conversation next time you see each other.
	3	Very true, if you both are just sniping from behind, for example, instead of just going up and being aggressive in a way, then you make a connection there.
**Living on social media**		
	3	Because there are, like, some people who just live on social media. It’s us who make the bloggers. We’re the ones saying what we want. And then they have to find a way to give it to us, sort of.
	3	I think maybe it has to do with... Maybe you’re a bit conflict avoidant and you’re worried about seeing, or, worried about how the other person will react and if you do it on social media, then you kind of don’t have to see the reaction.
	1	No, I mean that’s hard, but I think that... For me at least, I’ll admit that sometimes it can be easier to send a message than to say it face-to-face. That says something about me, too. But that the reaction, I mean, that the recipient can interpret it in another way, or in a bit of a different way, than it was meant is better than seeing the immediate reaction of the recipient, which might be different to what you hoped for, kind of.
	1	But in a way... When you say something wrong in reality then it gets taken more personally than online. I can’t explain why.
	1	From [when] you’re little you learn that you need to be careful about talking to people you don’t know. So, if you’ve met someone first, you know them in a way, and you feel safer. And then you chat on social media. Because then you have an idea of who the person is and you’re not tricked.
**Generation gap**		
	5	Yeah, when I communicate with a person who uses them, I kind of think that I don’t analyze it in the same way, because they’re a different generation, so I like adapt. But, I still don’t use smileys, I just do what I do, mostly, but I kind of adapt it, like I don’t write slang when I chat with mum, but when I chat with others and they use it I would either think it’s a typo or that it was, that there was something more behind it. So you wonder if it was on purpose.
	5	We’ve left Facebook because of parents. And now granny’s on there, as well.
	1	It’s good to keep things a bit separate. So, you’ve got different places for different relationships.
**Social norms and cross-border behaviors**		
	2	I guess the social norms we follow are less strict online.
	1	I would search for the person on Facebook. Check if we have friends in common. That’s the first thing I look at. Or I look at who follows the person on Instagram. If there’s no one...
	4	If they’re fake, they’re always fake online.
	4	Not to be rude, but I think it’s probably easier if you find it hard to talk to people face-to-face... Then this is something that will help you. But for most people real relations are worth more. If that’s ok to say.
	4	Something else I’ve noticed is that it’s people who have social problems who post their SnapChats on Facebook and say, “can you add me?” or “can I add you?” and ask for other people’s SnapChats. That’s understandable if people have social problems and want to find friends online. That’s when you post that on Facebook.
	4	There was a girl who suddenly started sending me messages saying “I’ve seen you at school”... That was a bit weird, I thought. And then she never stopped sending me messages. She asked me so many strange things. I don’t know. It was a bit scary.
	4	I feel like people ask like “hi, can we meet?” and you’ve never had anything to do with them before. Mostly you just try to ignore it, because you don’t know who they are or what it will be like.
	4	But maybe that’s why it’s kind of like... That’s why Tinder is looked down on maybe. As that’s a place you can be fooled much easier. Because some people go there to find comfort maybe.
**Self-agency and perceived control**		
	1	You kind of need to be in direct contact with the person. You can pretend to be anybody on SnapChat—the way you talk about everything. You can seem a lot more confident, etc.
	3	So, er, if you use the platforms Messenger and SnapChat, on Messenger you can choose when you want to reply, right. So if you get... Now he got a message. Of all things..., yeah. So, if you get a message on Messenger it’s, like, maybe a reply you need to think about a bit, I do that myself a lot. So, it’s like, you choose a bit like: “I don’t want to answer this yet, I know I’ve got a message, but I don’t need to answer yet” and then when you’re like kind of ready to answer, sounds a bit weird, but then yeah, you can answer. But in real life, it’s like, you kind of have to have a topic of conversation that you need to come up with then and there. But if you’re like online, and like Messenger, you can like come up with stuff bit by bit, right. Because then you have a lot of time to think about different things, kind of, and if you use SnapChat, then there’s that thing of having to answer right away. It’s normal if you snap someone, then you don’t like answer right after you get it, like. You wait maybe like the same amount of time as that person, the other person, waited to open and answer your snap, right? That’s like roughly how it goes, normally...
	1	But if you’re not sure, you have all these other social media you can check with. If there’s anyone on, for example, Tinder you think there’s something strange about, you can check on Instagram and see if you find anything. Or you can go on Instagram and see if there’s something there.
	1	No, I mean that’s hard, but I think that... For me at least, I’ll admit that sometimes it can be easier to send a message than to say it face-to-face. That says something about me, too. But that the reaction, I mean, that the recipient can interpret it in another way, or in a bit of a different way, than it was meant is better than seeing the immediate reaction of the recipient, which might be different to what you hoped for, kind of.
	1	When we started adding each other on SnapChat. I got her SnapChat and she got mine. And I didn’t start... The first thing I sent wasn’t, like, a picture of me. It was what I was doing and stuff. And then she did it, and I saw she was in high school, sat with her books and stuff.
	3	It’s easier to talk to people when you don’t get the signals from their faces and body... It’s kind of easier to talk to them when you don’t see how they react, like. And easier to talk also because you get a chance to think about what you’re talking about longer before you say it.
	2	Yeah, true. I feel like “friend” in real life, that’s more someone you can contact and be with. But friend on social media that doesn’t need to be people you even want to hang out with, it’s just like... Just to show that you know people!
**Mentalization**		
	1	I think both are just as genuine, just the process of messaging goes faster, because it’s easier to share since we kind of don’t see the response... We always want approval and that type of thing, and see that what we say and do is right.

**Table 2:** Field correspondence: themes included on Post-it notes, but not included in full-class or focus group discussions.

Theme	Post-it note content
Inspiration and shopping or fashion	Identify the latest in fashion A place where I find inspiration
News and knowledge acquisition	Find out what’s going on in the world on a daily basis Source of useful information
Social media as a sanctuary	A place where my parents can’t interfere so much A place where you can hide behind anonymity It is my identity without being judged A place where you hide how you really feel
New life	A place where you can create a new and different life Get to know people all over the world
Display differences in socioeconomic status	Display those who cannot afford an iPhone
Fake news	Information can be fake
Misunderstandings	Misunderstandings can easily arise due to lack of body language
Monitoring others	Easy way to monitor others A place where I study others
Stress and psychiatric problems	Can lead to loneliness, anxiety, and depression Social media kept me awake at night ­Social media leads to stress Feeling of being an outsider. Shows what your friends are doing without you
Social media dependence	May be all-consuming and lead to poor self-image It is a prison that makes us slaves because it is addictive Social media creates FOMO (fear of missing out)
Stops face-to-face communication	I spend a lot of time on Instagram. I stop talking to my friends. I’m just chatting
Cyberbullying and harassment	Social media leads to bullying Consequence of too few likes Easier to use nasty language online Nude pictures can be spread
Escalate body image issues and negative social comparison	Social media creates body image issues I compare myself with picture-perfect people, although I know the pictures are retouched

## Discussion

### Principal Findings

Social media provides distinct platforms for accommodating the core human need for being emotionally affiliated with a community [3]. Venturing beyond earlier models, which are solely concerned with face-to-face positions [10-14], four of the five suggested model positions are based on trajectories resulting from introducing social media to face-to-face social interaction. Hence, findings that primarily rely on face-to-face interactions are deficient when it comes to reflecting current social interfaces.

Reflecting previous research [40,41], similarities in behaviors, values, and interests seem to be consistent triggers for relationship formation and a perceived match between the preferred and actual social platform. This is particularly the case with regard to positions 1, 2, and 3. Also in line with previous research [15], mentalization or *the ability to understand the mental state, of oneself or others, that underlies overt behavior* [42], particularly during early phases of contact, seems to be a decisive capacity when it comes to validly evaluating social contexts, while social skills seem to be catalysts for contact attainment, both on social media and in face-to-face interactions. Another similarity between social media and face-to-face interactions is that perceived agency, which *encompasses the belief in the power of one’s own ability to affect outcome* [8], seems consistently correlated with social mastery, network development, and psychological well-being [8,43,44]. Thus, mastery of social media resembles mastery of face-to-face interactions and failings on social media appear similar to failings in face-to-face social settings. On this basis, we will argue that the perhaps most obvious point of departure for analysis, namely, to consider social media as the *new* (ie, the figure) and face-to-face interaction as the *point of reference* (ie, the background) is not the most fruitful approach. We propose that analyzing match and mismatch between preferred and actual social interaction platforms will result in a more valid analysis.

### Match Between Preferred and Actual Social Interaction Platforms

Individuals in positions 1 and 2 seem to achieve a match after a short and cost-effective social trajectory, reflecting a model starting point of face-to-face social interactions. These positions appear to involve well-established rituals and structures [45,46]. *Match* seems determined by whether the individuals seek the available social gains, master the current social norms, and provide a social functioning type that allows their needs to be fulfilled.

### The Vulnerable Position 3

Findings indicate a vulnerable transition for individuals in position 3. Although experiencing a fruitful match between preferred and actual social media platforms in late adolescence, these individuals seem to have had a social trajectory characterized by more fundamental contextual and social change. Transition value from earlier social life also seems lower compared to individuals in positions 1 and 2. Expected attendance on face-to-face arenas during adolescence [18,47], combined with accommodation of new social media game rules, seems to add additional stress for many. The aforementioned value-laden conflict between positions 2 and 3 on what authentic social interactions are may also cause stress. Stress associated with social interaction may in turn lead to increased social withdrawal from face-to-face interactions [48], yet without having established a robust social media network.

Social withdrawal is frequently used as a strategy to reduce acute social discomfort. However, as a long-term solution, withdrawal reinforces rather than solves social problems [48]. Anonymity, distance, and the emerging possibilities for complex emotional affiliation with an online community [2,25] may accelerate and cement social withdrawal from face-to-face interactions for individuals vulnerable to social distress. Hence, for individuals with match potential within positions 1 or 2, such a trajectory may result in a social *no-man’s land*, position 4, and mismatch. Early position 3 affiliation may thus predict increased risk for social challenges and psychological problems [1,49]. Individuals suffering from early face-to-face social defeats or adverse events, such as bullying [20,50], seem particularly vulnerable to this route.

Conversely, individuals that are well-established in position 3 indicate that social media provides new and appealing social affiliation opportunities not available pre-social media [2,12,24,51].

### The Matthew Effect

Establishing social skills and associated capacities, such as self-agency and mentalization, may in simple terms be understood through a paraphrase on the Matthew effect: those who master become better; those who fail, fail again. Individuals who, in early age, experience mastering social contexts will have a higher chance of establishing a feeling of self-agency, better social skills, and, in time, the ability to mentalize [8,15]. Findings indicate that this momentum created by mastering and building social skills may predict mastering either face-to-face, on social media, or both. The associated increase in mentalization corresponds to the finding that mentalization ability is particularly important for valid early relational evaluation [15] and increases the likelihood of picking a social platform corresponding to personal characteristics and needs. Echoing previous research, such development is associated with flexible psychological strategies, well-functioning relationships, and psychological well-being [3,15,52]. Findings indicate that these positive effects are prominent in all established matched positions.

Conversely, findings suggest that early failure in mastering face-to-face social skills lowers the chance of establishing self-agency, social skills, and mentalization. Individuals to whom this applies also seem to lack social triggers, including behaviors, values, or interests that they share with others [40,41], thus reducing the chances of relationship formation and social mobility. Findings indicate that this lack of social momentum is associated with poor mastering of face-to-face and, in time, social media interaction. This pattern also seems associated with decreased chances for achieving a match between preferred and actual type of social platform and, ultimately, social rejection from others [53]. Thus, instead of psychological well-being, these individuals may experience increased stress [54], anxiety, and *learned helplessness* [48,55,56], leading to passivity and withdrawal from social encounters. Findings indicate that this pattern is self-reinforcing, continuously increasing the distance between the person and the social mainstream. These continuous social defeats and unpleasant psychological conditions, combined with the core hunt for social affiliation, may partly explain why some individuals over time seek different online echo chambers rather than a matched position.

### Implications

Model position status has the potential to predict human behavior and is, therefore, relevant for disciplines such as internet and gaming research, youth research, and mental health research, as well as for practitioners. Individuals in all established matched positions seem robust, except individuals in early position 3, who seem vulnerable.

Position 4 may predict psychopathology [2,57,58]. General limitations in social skills, self-agency, and mentalization, but plausibly also poor insight into how their own behaviors affect others, make these individuals particularly vulnerable for long-term social exclusion. Further, social media’s unlimited number of possible contacts and few boundaries related to time and space [12,27,59] may reduce social correction and instead facilitate a continuation of a negative social trajectory. Position 5 may also predict psychopathology. Individuals in this position do not necessarily have poor insight, but have problems transferring from actual to preferred social platform. This may apply to individuals living in institutions and forced settings, for example, prison populations.

A detailed model of social interaction including new media platforms provide resources for measure development within health science. For instance, items targeting social functioning in widely used measures for psychiatric diagnoses do not include social media functioning [14], even if people withdrawing from social contact as part of mental illness development lead active social media lives [60]. This paradox points to a latency in measure development in a social reality marked by rapid technology developments. Research-based operationalizations of social media functions may inform necessary development.

### Limitations

The general limitations of qualitative research apply to this investigation: findings are context dependent and pertain to the participants and setting in which the study was conducted. There was an overrepresentation of females (71%), the population was selected from individuals attending school, and interviews were conducted face-to-face and only in Norway. Also, data were not used to analyze how specific demographic or other characteristics might influence the participants’ social interactions, differences in their perceptions, and overall attitude. All these factors affect generalizability.

To accommodate some of the limitations associated with qualitative methodology, we performed a field-correspondence technique using Post-it notes. Although expected themes, such as pornography habits, were omitted, participants reported more personally sensitive themes in this format (see Table 2). These dealt, in particular, with the negative sides of using social media.

### Future Research

The overarching goal for future research is to explore and develop the validity of the proposed model, and to investigate to what degree falling into one position is associated with positive (ie, mastery and protection) and negative health and well-being.

**Figure 3:**
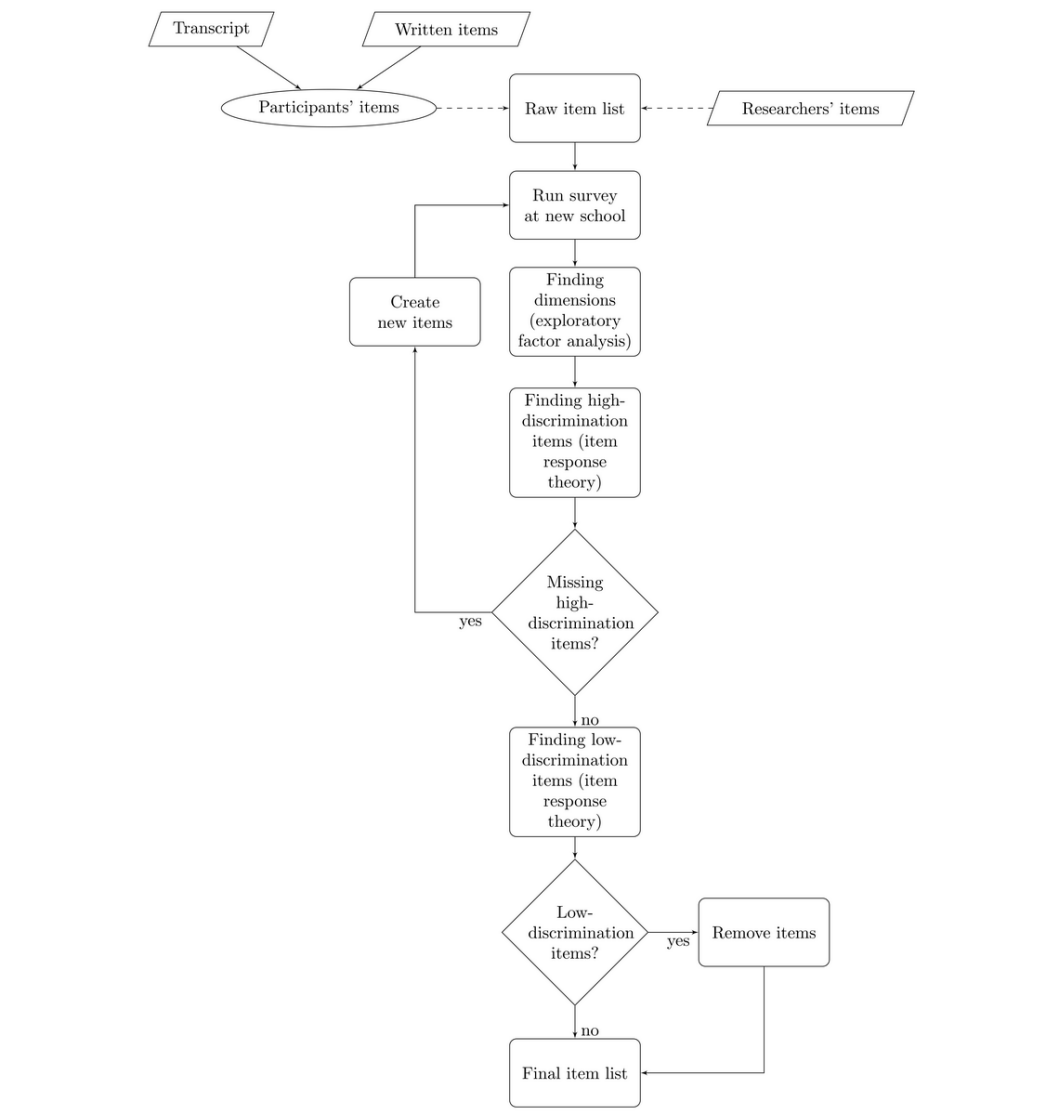
Model validation procedure.

We will test this model with a large-scale quantitative survey design, as presented in Figure 3. The initial survey items will be drawn from suggestions written by participants in this study. These items will be supplemented with questions derived from the focus groups, class sessions, and items made by the researchers. This initial large pool of items will be used in the initial large-scale survey. Using exploratory factor analysis or similar, the questions will be divided into different dimensions. Using item response theory, items with low discrimination will be excluded. Item response theory will also be used to highlight ranges in the different dimensions where the survey is missing high-discrimination items. This framework can be used to generate testable hypotheses and items on dimensions of social interaction. To study context-specificity and generalizability, we propose that this research procedure could be implemented across different countries and contexts, and we welcome collaboration.

## References

[1] Riehm KE, Feder KA, Tormohlen KN, Crum RM, Young AS, Green KM, Pacek LR, La Flair LN, Mojtabai R (2019). Associations between time spent using social media and internalizing and externalizing problems among US youth. JAMA Psychiatry.

[2] Kaplan AM, Haenlein M (2010). Users of the world, unite! The challenges and opportunities of social media. Bus Horiz.

[3] Thoits PA (2011). Mechanisms linking social ties and support to physical and mental health. J Health Soc Behav.

[4] (2019). Statista.

[5] Lenhart A, Duggan M, Perrin A, Stepler R, Rainie L, Parker K (2015). Pew Research Center.

[6] Children’s Commissioner’s Office, Revealing Reality (2017). Life in ‘Likes’: Children’s Commissioner Report Into Social Media Use Among 8-12 Year Olds.

[7] The Lancet (2018). Children and social media. Lancet.

[8] Bandura A (1982). Self-efficacy mechanism in human agency. Am Psychologist.

[9] Ajzen I, Kuhl J, Beckmann J (1985). From intentions to actions: A theory of planned behavior. Action Control: From Cognition to Behavior.

[10] Koteyko N, Hunt D, Gunter B (2015). Expectations in the field of the internet and health: An analysis of claims about social networking sites in clinical literature. Sociol Health Illn.

[11] Hercheui MD (2011). A literature review of virtual communities. Inf Commun Soc.

[12] Lee EWJ, Ho SS, Lwin MO (2016). Explicating problematic social network sites use: A review of concepts, theoretical frameworks, and future directions for communication theorizing. New Media Soc.

[13] Litt E (2013). Measuring users’ internet skills: A review of past assessments and a look toward the future. New Media Soc.

[14] Bjornestad J, Hegelstad WTV, Berg H, Davidson L, Joa I, Johannessen JO, Melle I, Stain HJ, Pallesen S (2019). Social media and social functioning in psychosis: A systematic review. J Med Internet Res.

[15] Fonagy P, Gergely G, Jurist EL (2018). Affect Regulation, Mentalization, and the Development of the Self.

[16] Vygotsky LS (1980). Mind in Society: The Development of Higher Psychological Processes.

[17] Muuss RE (1988). Theories of Adolescence. 5th edition.

[18] Blakemore SJ (2018). Inventing Ourselves: The Secret Life of the Teenage Brain.

[19] Griffiths MD (2011). Internet sex addiction: A review of empirical research. Addict Res Theory.

[20] Ehman AC, Gross AM (2019). Sexual cyberbullying: Review, critique, & future directions. Aggress Violent Behav.

[21] Jenaro C, Flores N, Frías CP (2018). Systematic review of empirical studies on cyberbullying in adults: What we know and what we should investigate. Aggress Violent Behav.

[22] Fisher BW, Gardella JH, Teurbe-Tolon AR (2016). Peer cybervictimization among adolescents and the associated internalizing and externalizing problems: A meta-analysis. J Youth Adolesc.

[23] Allen JP, Land D, Cassidy J, Shaver PR (1999). Attachment in adolescence. Handbook of Attachment: Theory, Research, and Clinical Applications.

[24] Jasanoff S (2016). The Ethics of Invention: Technology and the Human Future.

[25] Kuss DJ, Griffiths MD (2011). Online social networking and addiction: A review of the psychological literature. Int J Environ Res Public Health.

[26] Spinzy Y, Nitzan U, Becker G, Bloch Y, Fennig S (2012). Does the internet offer social opportunities for individuals with schizophrenia? A cross-sectional pilot study. Psychiatry Res.

[27] Troiano G, Nante N (2018). Emoji: What does the scientific literature say about them? A new way to communicate in the 21st century. J Hum Behav Soc Environ.

[28] Wang Y, McKee M, Torbica A, Stuckler D (2019). Systematic literature review on the spread of health-related misinformation on social media. Soc Sci Med.

[29] Braun V, Clarke V (2019). Reflecting on reflexive thematic analysis. Qual Res Sport Exerc Health.

[30] Braun V, Clarke V (2006). Using thematic analysis in psychology. Qual Res Psychol.

[31] Fasting RB (2013). Adapted education: The Norwegian pathway to inclusive and efficient education. Int J Inclusive Educ.

[32] Braun V, Clarke V (2019). To saturate or not to saturate? Questioning data saturation as a useful concept for thematic analysis and sample-size rationales. Qual Res Sport Exerc Health.

[33] Yeager DS, Romero C, Paunesku D, Hulleman CS, Schneider B, Hinojosa C, Lee HY, O'Brien J, Flint K, Roberts A, Trott J, Greene D, Walton GM, Dweck CS (2016). Using design thinking to improve psychological interventions: The case of the growth mindset during the transition to high school. J Educ Psychol.

[34] Brown T (2008). Design thinking. Harv Bus Rev.

[35] Brown T, Wyatt J (2010). Design thinking for social innovation. Dev Outreach.

[36] Hill CE, Knox S, Thompson BJ, Williams EN, Hess SA, Ladany N (2005). Consensual qualitative research: An update. J Couns Psychol.

[37] Reisdorf BC, Groselj D (2015). Internet (non-)use types and motivational access: Implications for digital inequalities research. New Media Soc.

[38] Brody N (2018). Opting out of social media: Online communication attitudes mediate the relationship between personality factors and Facebook non-use. South Commun J.

[39] Hargittai E, Piper AM, Morris MR (2018). From internet access to internet skills: Digital inequality among older adults. Univers Access Inf Soc.

[40] Bornstein RF (1989). Exposure and affect: Overview and meta-analysis of research, 1968–1987. Psychol Bull.

[41] Zajonc RB (1968). Attitudinal effects of mere exposure. J Pers Soc Psychol.

[42] Fonagy P, Bateman AW (2006). Mechanisms of change in mentalization-based treatment of BPD. J Clin Psychol.

[43] Jeannerod M (2009). The sense of agency and its disturbances in schizophrenia: A reappraisal. Exp Brain Res.

[44] McAdams DP (2020). Psychopathology and the self: Human actors, agents, and authors. J Pers.

[45] Bourdieu P, Grusky DB, Szelenyi S (2018). Cultural reproduction and social reproduction. Inequality: Classic Readings in Race, Class, and Gender.

[46] Goffman E (1990). The Presentation of Self in Everyday Life.

[47] Willoughby M (2018). A review of the risks associated with children and young people’s social media use and the implications for social work practice. J Soc Work Pract.

[48] Gazelle H, Rubin KH (2019). Social withdrawal and anxiety in childhood and adolescence: Interaction between individual tendencies and interpersonal learning mechanisms in development: Introduction to the special issue. J Abnorm Child Psychol.

[49] McCrae N, Gettings S, Purssell E (2017). Social media and depressive symptoms in childhood and adolescence: A systematic review. Adolesc Res Rev.

[50] Franklin P, Hossain R, Coren E (2016). Social media and young people’s involvement in social work education. Soc Work Educ.

[51] Verduyn P, Ybarra O, Résibois M, Jonides J, Kross E (2017). Do social network sites enhance or undermine subjective well-being? A critical review. Soc Issues Policy Rev.

[52] Zeegers MAJ, Colonnesi C, Stams GJM, Meins E (2017). Mind matters: A meta-analysis on parental mentalization and sensitivity as predictors of infant-parent attachment. Psychol Bull.

[53] Becker HS (1963). Outsiders: Studies in the Sociology of Deviance.

[54] Beyens I, Frison E, Eggermont S (2016). “I don’t want to miss a thing”: Adolescents’ fear of missing out and its relationship to adolescents’ social needs, Facebook use, and Facebook related stress. Comput Human Behav.

[55] Seligman ME (1972). Learned helplessness. Annu Rev Med.

[56] Seligman MEP (2006). Learned Optimism: How to Change Your Mind and Your Life.

[57] van Os J, Kenis G, Rutten BPF (2010). The environment and schizophrenia. Nature.

[58] Gunderson JG, Herpertz SC, Skodol AE, Torgersen S, Zanarini MC (2018). Borderline personality disorder. Nat Rev Dis Primers.

[59] Lips AMB, Eppel EA (2016). Understanding and explaining online personal information-sharing behaviours of New Zealanders: A new taxonomy. Inf Commun Soc.

[60] Seabrook EM, Kern ML, Rickard NS (2016). Social networking sites, depression, and anxiety: A systematic review. JMIR Ment Health.

